# EMX2 Is a Predictive Marker for Adjuvant Chemotherapy in Lung Squamous Cell Carcinomas

**DOI:** 10.1371/journal.pone.0132134

**Published:** 2015-07-01

**Authors:** Dongsheng Yue, Hui Li, Juanjuan Che, Yi Zhang, Bhairavi Tolani, Minli Mo, Hua Zhang, Qingfeng Zheng, Yue Yang, Runfen Cheng, Joy Q. Jin, Thomas W. Luh, Cathryn Yang, Hsin-Hui K. Tseng, Etienne Giroux-Leprieur, Gavitt A. Woodard, Xishan Hao, Changli Wang, David M. Jablons, Biao He

**Affiliations:** 1 Department of Lung Cancer, Lung Cancer Center, Tianjin Medical University Cancer Institute and Hospital, National Clinical Research Center of Cancer, Key Laboratory of Cancer Prevention and Therapy, Tianjin 300060, China; 2 Thoracic Oncology Program, Department of Surgery, Helen Diller Family Comprehensive Cancer Center, University of California San Francisco, San Francisco, CA 94115, United States of America; 3 Department of Oncology, Beijing Friendship Hospital of Capital Medical University, Beijing 100050, China; 4 Department of Thoracic Surgery, Xuanwu Hospital, Capital Medical University, Beijing 100053, China; 5 School of Life Sciences, Tsinghua University, Beijing 10084, China; 6 Key Laboratory of Carcinogenesis and Translational Research (Ministry of Education), Thoracic Surgery II, Peking University Cancer Hospital & Institute, Beijing 100142, China; 7 Department of Pathology, Lung Cancer Center, Tianjin Medical University Cancer Institute and Hospital, Tianjin 300060, China; University of Algarve, PORTUGAL

## Abstract

**Background:**

Squamous cell carcinomas (SCC) account for approximately 30% of non-small cell lung cancer (NSCLC). Current staging methods do not adequately predict outcome for this disease. EMX2 is a homeo-domain containing transcription factor known to regulate a key developmental pathway. This study assessed the significance of EMX2 as a prognostic and predictive marker for resectable lung SCC.

**Methods:**

Two independent cohorts of patients with lung SCC undergoing surgical resection were studied. EMX2 protein expression was examined by immunohistochemistry, Western blot, or immunofluorescence. EMX2 expression levels in tissue specimens were scored and correlated with patient outcomes. Chemo-sensitivity of lung SCC cell lines stably transfected with EMX2 shRNAs to cisplatin, carboplatin, and docetaxel was examined *in vitro*.

**Results:**

EMX2 expression was down-regulated in lung SCC tissue samples compared to their matched adjacent normal tissues. Positive EMX2 expression was significantly associated with improved overall survival in stage I lung SCC patients, and in stage II/IIIA lung SCC patients receiving adjuvant chemotherapy. EMX2 expression was also associated with expression of EMT markers in both lung SCC cell lines and tissue samples. Knock-down of EMX2 expression in lung SCC cells promoted chemo-resistance and cell migration.

**Conclusions:**

EMX2 expression is down-regulated in lung SCC and its down-regulation is associated with chemo-resistance in lung SCC cells, possibly through regulation of Epithelial-to-Mesenchymal Transition (EMT). EMX2 may serve as a novel prognostic marker for stage I lung SCC patients and a prediction marker for stage II/IIIA lung SCC patients receiving adjuvant chemotherapy.

## Introduction

Lung cancer is the most common cancer worldwide and the leading cause of cancer-related mortality [1]. Approximately 85% of newly diagnosed lung cancers are non—small cell lung cancer (NSCLC). Adenocarcinoma and squamous cell carcinoma (SCC) are the most frequent histologic subtypes, accounting for 50% and 30% of NSCLC cases, respectively [2]. Despite advances in our knowledge in lung tumor biology and improvements in surgery, radiotherapy and chemotherapy, we have made little impact on the overall survival rate of NSCLC patients in the past 30 years, indicating that our current staging methods are not adequate in predicting outcome [3]. Current development efforts in prognostic and predictive biomarkers are being made to improve staging of lung cancers but these are mostly for adenocarcinomas [4–8]. However, such studies for lung SCC are limited [9–11]. Therefore, there is an urgent need to identify novel prognostic/predictive markers for lung SCC, which has unique clinic-pathological and molecular characteristics.

EMX2 (empty spiracles homeobox 2), a human homolog of the Drosophila empty spiracles gene (*ems*), is shown to regulate the Wnt pathway during development. The homeobox gene family encodes transcription factors, regulating morphogenesis and cell differentiation during embryogenesis by activating or repressing the expression of target genes [12]. In addition to its important role in embryologic development, several studies suggest that EMX2 may be involved in human tumorigenesis [13–16]. More recently, we have demonstrated epigenetic silencing of EMX2 expression in lung adenocarcinomas and its association with poor clinical outcomes in early stage lung adenocarcinoma patients [17, 18]. In this study, we hypothesized that EMX2 might also serve as a novel prognostic and/or predictive biomarker in lung SCC. Our aim was to determine whether there was a correlation between EMX2 expression and overall 5-year survival as a primary endpoint in patients with early to mid-stage lung SCC.

Since most patients with lung cancer receive chemotherapy, we sought to determine if EMX2 expression is predictive of response to chemotherapy *in vitro*. Thus, a secondary endpoint was to evaluate the correlation between EMX2 expression and cell survival in chemo-treated cells lines. Further, in spite of chemotherapeutic intervention, lung SCC tends to be locally aggressive [19]. Epithelial to mesenchymal transition (EMT) enables cancer cells to invade surrounding tissues and generate distant metastases [20], and has been shown to be associated with poor prognosis and chemo-resistance in different tumor models [21–23]. In this study, we also investigated possible molecular mechanism relations between EMX2 and EMT in lung SCC.

## Materials and Methods

### Ethics Statement

This investigation has been conducted in accordance with ethical standards and according to the Declaration of Helsinki, as well as national and international guidelines. Patients consented, in writing, to tissue specimen collection prospectively, and the study was approved by the Research Ethics Committee of Cancer Institute and Hospital of Tianjin Medical University, Beijing Cancer Hospital, as well as the University of California, San Francisco Institutional Review Board on Human Research.

### Patients

Patients with stage I-IIIA lung SCC undergoing complete pulmonary resection and systematic node dissection of the hilar and mediastinal lymph nodes at the Cancer Institute and Hospital of Tianjin Medical University (Cohort-1, 177 patients from 2004 to 2008) and Beijing Tumor Hospital (Cohort-2, 42 patients from 1995 to 2000) were entered into the study (Table 1). In addition to Cohorts 1 and 2, we used a different set of 16 matched pairs of lung SCC and their adjacent normal tissue specimens banked at the time of surgical resection at the University of California, San Francisco (UCSF). Patients receiving neo-adjuvant chemotherapy or radiation therapy, or having a second primary cancer diagnosed within 5 years, were excluded from this study. Pathology of each tumor was diagnosed by two certified pathologists. TNM staging was performed according to the International System for Staging Lung Cancer pathologic classification system. Information on clinical variables and patient follow-up was extracted from a prospectively maintained patient database. Adjuvant chemotherapy used to treat patients with lung SCC were vinorelbine, paclitaxel, or gemcitabine plus carboplatin or cisplatin for Cohort-1, and vinorelbine, cyclophosphamide, or doxorubicin plus cisplatin for Cohort-2. The primary endpoint was overall survival (OS) with the time range from 3 to 84 months. EMX2 protein expression was measured by Western blot in 16 patients who had paired lung SCC as well as matched adjacent normal tissue banked at the time of surgical resection at UCSF. Results of Western Blot were confirmed with IHC. EMX2 staining was scored (0 for negative and 1–3 for positive) by a pathologist using a microscope.

**Table 1:** Patient characteristics and five-year overall survival (OS).

Characteristics	Cohort 1		Cohort 2	
	No.	5-yr OS (%)	No.	5-yr OS (%)
**Age (Years)**				
<60	71	40.10%	22	52.40%
≥60	106	59.90%	20	47.60%
**Gender**				
Male	151	85.30%	36	85.70%
Female	26	14.70%	6	14.30%
**Smoking history**				
Never	29	16.40%	10	16.40%
Smoker	148	83.60%	32	83.60%
**Surgical Procedure**				
Lobectomy	143	80.80%	32	76.20%
Pneumonectomy	30	16.90%	8	19.00%
Extended	4	2.30%	2	4.80%
**T stage**				
T1	45	25.40%	4	9.50%
T2	107	60.50%	10	23.80%
T3	25	14.10%	28	66.70%
**N stage**				
N0	126	71.20%	0	0%
N1	16	9.00%	24	57.10%
N2	35	19.80%	18	42.90%
**TNM Stage**				
I	91	51.40%	0	0%
II	48	27.10%	8	19%
IIIA	38	21.50%	34	81%
**Adjuvant chemotherapy (II-IIIA)**				
Yes	56	65.10%	30	71.40%
No	30	34.90%	12	28.60%
**EMX2**				
Positive	125	70.60%	28	66.70%
Negative	52	29.40%	14	33.30%

### Immunohistochemistry (IHC)

Immunohistochemical staining was performed using standard procedures. Primary antibodies used in the study included rabbit anti-human EMX2 (1:400; Pierce), rabbit anti-human E-cadherin (1:200; Cell Signaling), mouse anti-human and β-catenin (1:200; Millipore). Five micron tissue slides from tumor and adjacent normal lung tissue were de-paraffinized using xylene. Heat-mediated antigen retrieval was performed using citrate buffer (BioGenex Laboratories). Antibody staining was visualized with DAB (Histostain Plus Broad Spectrum, Invitrogen) and hematoxylin counterstain (Fisher Scientific). Representative fields were photographed using a Leica SCN400 slide scanner (Leica, Germany), and examined for positive nuclear staining. The mean number of positive cells in each tumor and matched normal tissue sample was compared using a two-tailed paired *t*-test. IHC staining of EMX2 was scored by two independent pathologists who were not aware of corresponding clinical information. Briefly, an IHC score was assigned by a combination of staining intensity (no staining = 0, light yellow staining = 1, yellowish brown staining = 2, strong brown staining = 3) and the percentage of positively stained cells (<25% = 1, 26–50% = 2, >51% = 3). A score of 0 was defined as negative, and a score of 1, 2, or 3 was defined as positive.

### Cell Culture

Lung SCC cell lines (H1703 and H2170) were purchased from American Type Culture Collection (ATCC). Both cell lines were cultured in RPMI 1640 (Life Technologies) supplemented with 10% fetal bovine serum (FBS), penicillin (100 IU/ml)/streptomycin (100 μg/ml) at 37°C in a humid incubator with 5% CO_2_.

### Transfection and RNA Interference

pcDNA 3.1/EMX2 mammalian expression-vector was subcloned from pCMV6-XL5/EMX2 vector (Origene). EMX2 shRNAs and control (non-silencing) shRNA (all are in pRFP-C-RS vector) were purchased from Origene. The targeted EMX2 sequences are: 5’-TCAAGCCATTTACCAGGCTTCGGAGGAAG-3’ and 5’-CGGTGGAGAATCGCCACCAAGCAGGCGAG-3’. Cells were plated in six-well plates with fresh media without antibiotics for 24 hours before transfection. Transfection was performed using Lipofectamine2000 (Invitrogen) according to the manufacturer’s protocol. Transfected cells were then re-plated in 10 cm dishes for selection with G418 (500 μg/ml; Invitrogen). Stable transfectants were maintained in regular medium with G418 (300 μg/ml) for further analysis.

### Proliferation Assay

CellTiter 96 Aqueous Proliferation Assay Kit (Promega) was used to determine cell proliferation according to the manufacturer’s protocol. The assays were performed in triplicate after 5×10^3^ cells/well were plated into 96-well plates and treated with cisplatin, carboplatin or docetaxel at different doses for 72 hours.

### Wound-healing Assay

Cells were prepared in 6-well plates and wounds were created by manually scraping the cell monolayer with a p-200 pipet tip. Cells were then incubated for 24 hours before images were acquired through a light microscope. To quantify the migration, four wounds were made for each condition, and cell migration was presented by the average of distance differences between 24hr and 0hr. Cell proliferation at 24hr was also monitored, and no significant difference was observed. All experiments have been conducted more than three times, and representative results were included in the text.

### Immunofluorescence Staining (IF)

Cells were seeded 24 hours before IF staining on 8-well chamber slides. Cells were fixed with 4% paraformaldehyde for 10 min at -20°C, permeabilized with 0.5% Triton X-100 for 10 min and blocked in 10%FBS/PBS for 1hour at room temperature, followed by overnight incubation with primary antibodies anti-EMX2 (1:400; Pierce), anti-E-Cadherin (1:200; Cell Signaling) and anti-β-catenin (1:100; BD)) at 4°C. After washing with PBS, cells were incubated with secondary Alexa488-conjugated IgG for 1 hour at room temperature. Slides were mounted in VectorShield mounting media with DAPI (Vector), and images were acquired with a Leica SCN400 slide scanner (Leica) and analyzed at 200× magnification.

### Western Blotting

Total proteins were extracted from cultured cells with M-PER extraction solution (Pierce), and from frozen tissues with T-PER extraction solution (Pierce). Western blots were performed following standard procedures. Antibodies applied to detect protein expressions were anti-EMX2 (1:500; Pierce), anti-N-Cadherin (1:500; Santa Cruz Technologies), anti-Vimentin (1: 20000; Abcam), anti-Snail (1:2000; Cell Signaling), anti-SLUG (1:250; Abcam) and anti-Actin (1:5000; Sigma).

### Statistical Analysis

The Kaplan-Meier method was used to estimate overall survival (OS). Differences in survival between the low-risk group (positive EMX2 IHC staining) and high-risk group (negative EMX2 IHC staining) were analyzed by a log-rank test. The associations between EMX2 positive/negative staining and discrete clinical factors were analyzed by the *t*-test, ANOVA with Bonferroni/Dunn test, Mann-Whitney’s U-test for variables with two-categories, and the Kruskal-Wallis test for variables with more than two categories. A Kappa test was used to evaluate the association between the expressions of EMX2 and EMT markers. IHC scores of 1–3 were grouped as positive “+”, and 0 was grouped as negative “-”. All analyses were done using the SPSS software package, version IBM SPSS Statistics 18.0. All reported *p*-values were two-sided. A *p* value of 0.05 or less was considered to be significant.

### Microarray Analysis

H2170 cells were stably transfected with EMX2 or empty vector control. Total RNA was extracted using Qiagen’s RNeasy Mini Kit. Total RNA quality was assessed using a Pico Chip on an Agilent 2100 Bioanalyzer (Agilent, Santa Clara, CA, USA). RNA was amplified and labeled with Cy3-CTP or Cy5-CTP using the Agilent low RNA input fluorescent linear amplification kits following the manufacturer’s protocol. Labeled cRNA was assessed using the Nandrop ND-100, and equal amounts of Cy3- and Cy5-labeled target were hybridized to Agilent whole-human genome 44 K ink-jet arrays. Arrays were scanned using the Agilent microarray scanner and raw signal intensities were extracted with Agilent Feature Extraction software. Genes that exhibited more than 2 fold expression changes were analyzed with the Functional Annotation Clustering Tool of David Bioinfornatics Database by comparing with the Gene Ontology (GO) of the KEGG_Pathway database. The DAVID Functional Annotation Clustering uses a novel algorithm to measure relationships among the annotation terms based on the degrees of their co-association genes to group the similar, redundant, and heterogeneous annotation contents from the same or different resources into annotation groups. This reduces the burden of associating similar redundant terms and makes the biological interpretation more focused in a group level. Enrichment scores of annotation clusters and EASE score (a modified Fisher Exact *p*-value) were utilized to describe enrichment. The higher the Enrichment scores, the more enriched. The smaller the EASE scores, the more enriched. Usually a *p*-value equal or smaller than 0.05 is to be considered strongly enriched in the annotation categories. The microarray data was deposited in the MUSC DNA Microarray Database (Record No.: _1409386959.676282).

## Results

### EMX2 expression is down-regulated in lung SCC

**Fig 1:**
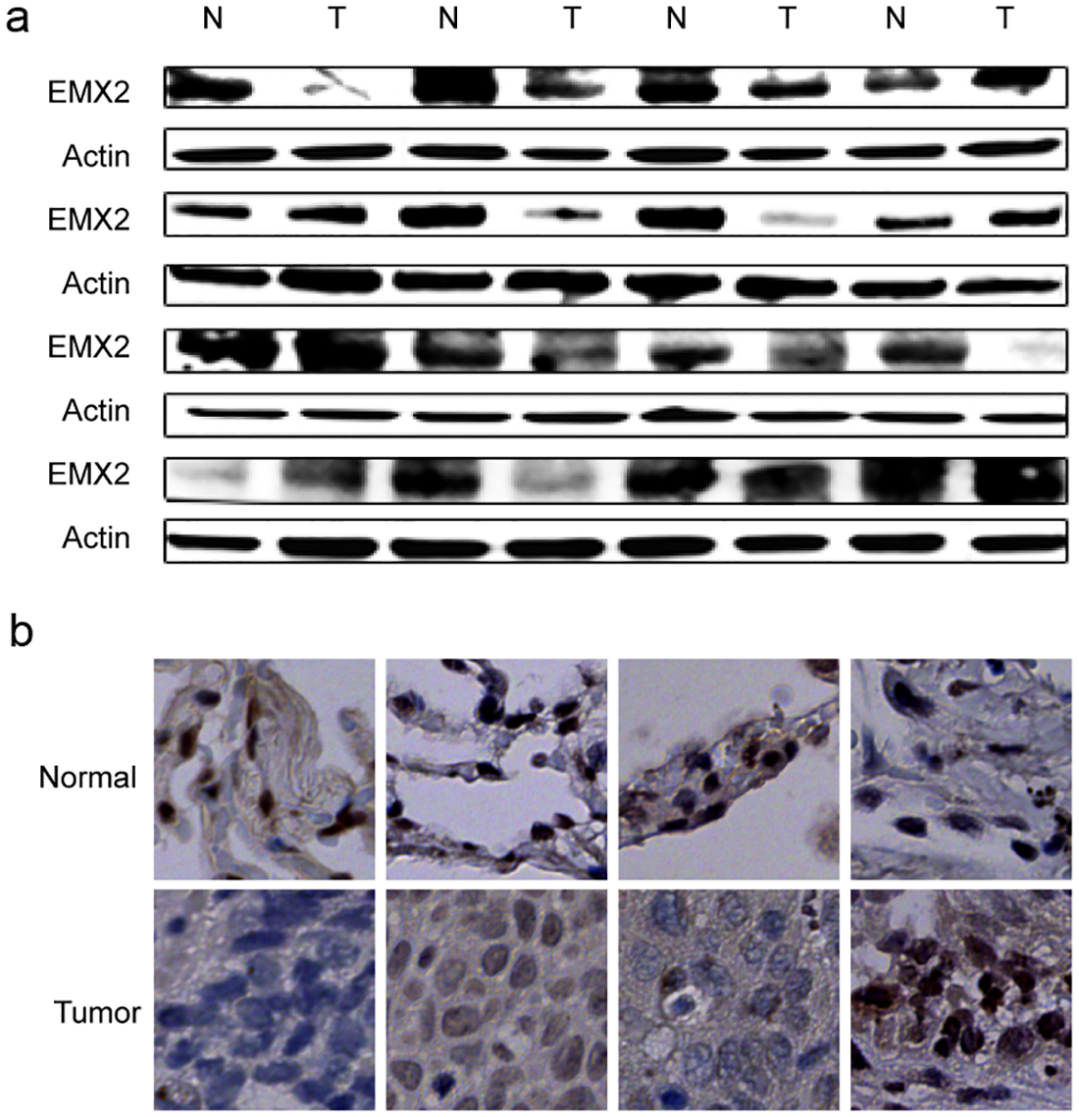
EMX2 is down-regulated in lung SCC tissue samples. (a) Western blots of EMX2 protein expression in sixteen matched pairs of lung SCC tissue samples. Actin served as a loading control. (b) IHC staining of EXM2 expression in matched pairs of lung SCC specimens. The four pairs of tissue samples shown were from the same patients as shown in the first row of Western blot in panel a.

We first examined EMX2 protein expression in 16 patients who had paired lung SCC as well as matched adjacent normal tissue banked at the time of surgical resection at UCSF. Ten of 16 lung SCC specimens (62.5%) expressed less EMX2 protein than their matched adjacent normal tissues (Fig 1a). To confirm these results, we examined EMX2 expression levels of the first set of 4 pairs of tissue samples shown in Fig 1a and observed similar results (Fig 1b). Further, we observed nuclear localization of EMX2 in those specimens and found EMX2 protein down-regulation in the tumor tissues when compared to their matched normal tissues, consistent with our previous results.

### EMX2 expression is associated with improved survival in stage I lung SCC patients

**Fig 2:**
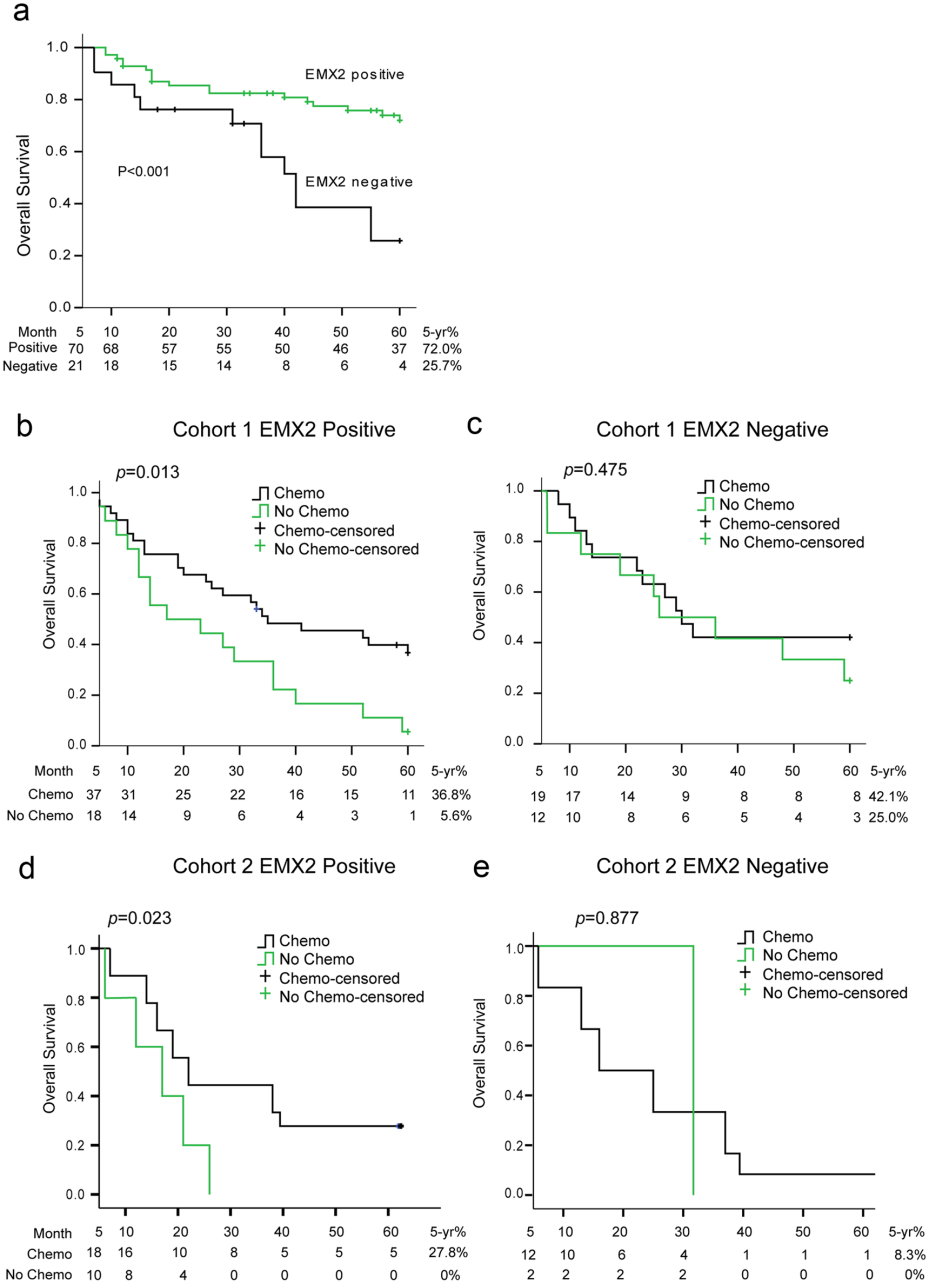
EMX2 expression is associated with clinical outcomes. (a) Kaplan-Meier plot of overall survival by EMX2 expression in 91 patients with stage I lung SCC in Cohort-1. (b-e) Kaplan-Meier plots of overall survival by IHC staining of EMX2 in patients with stage II-IIIA lung SCC in Cohort-1 (panels b and c) and Cohort-2 (panels d and e).

We next assessed EMX2 protein expression by IHC using tissue arrays of lung SCC specimens (clinical characteristics of the patients were summarized in Table 1). EMX2 staining was scored (0 for negative and 1–3 for positive) by a pathologist using a microscope. None of the 91 stage I lung SCC patients in Cohort-1 received neo-adjuvant chemotherapy before surgery. Kaplan-Meier analysis using Cox proportional hazards modeling demonstrated a strong association between EMX2 protein expression and overall survival in stage I lung SCC patients (Fig 2a). Overall survival was significantly better in the patients with positive EMX2 staining compared to those with negative EMX2 staining (*p < 0*.*001*; 5-year OS: 72.0% vs. 25.7%;). A statistically significant difference in overall survival was not observed in stage II/IIIA lung SCC patients (*p = 0*.*482*) (data not shown).

### EMX2 expression is associated with improved survival in stage II/IIIA lung SCC patients receiving adjuvant chemotherapy

Approximately 65% (56 of 86) patients with stage II/IIIA lung SCC in Cohort-1 received adjuvant chemotherapy after surgical intervention (Table 1). In the patients with positive EMX2 staining, we found that overall survival for the patients who received adjuvant chemotherapy was significantly better than that for patients who did not (Fig 2b; 5-year OS: 36.8% vs. 5.6%; *p* = 0.013). In the patients with negative EMX2 staining, however, overall survival did not differ between those who received adjuvant chemotherapy and those who did not (Fig 2c; 5-year OS: 42.1% vs. 25.0%; *p* = 0.475). Moreover, we validated our observations in an independent cohort of stage II/IIIA lung SCC patients (Cohort-2) in a double-blinded manner. In the patients with positive EMX2 staining of Cohort-2, overall survival for the patients who received adjuvant chemotherapy was also significantly better than that for patients who did not (Fig 2d; 5-year OS: 27.8% vs. 0%; *p* = 0.023). In the patients with negative EMX2 staining of Cohort-2, no difference in overall survival was observed between those who received adjuvant chemotherapy and those who did not (Fig 2e; 5-year OS: 8.3% vs. 0%; *p* = 0.877).

**Fig 3:**
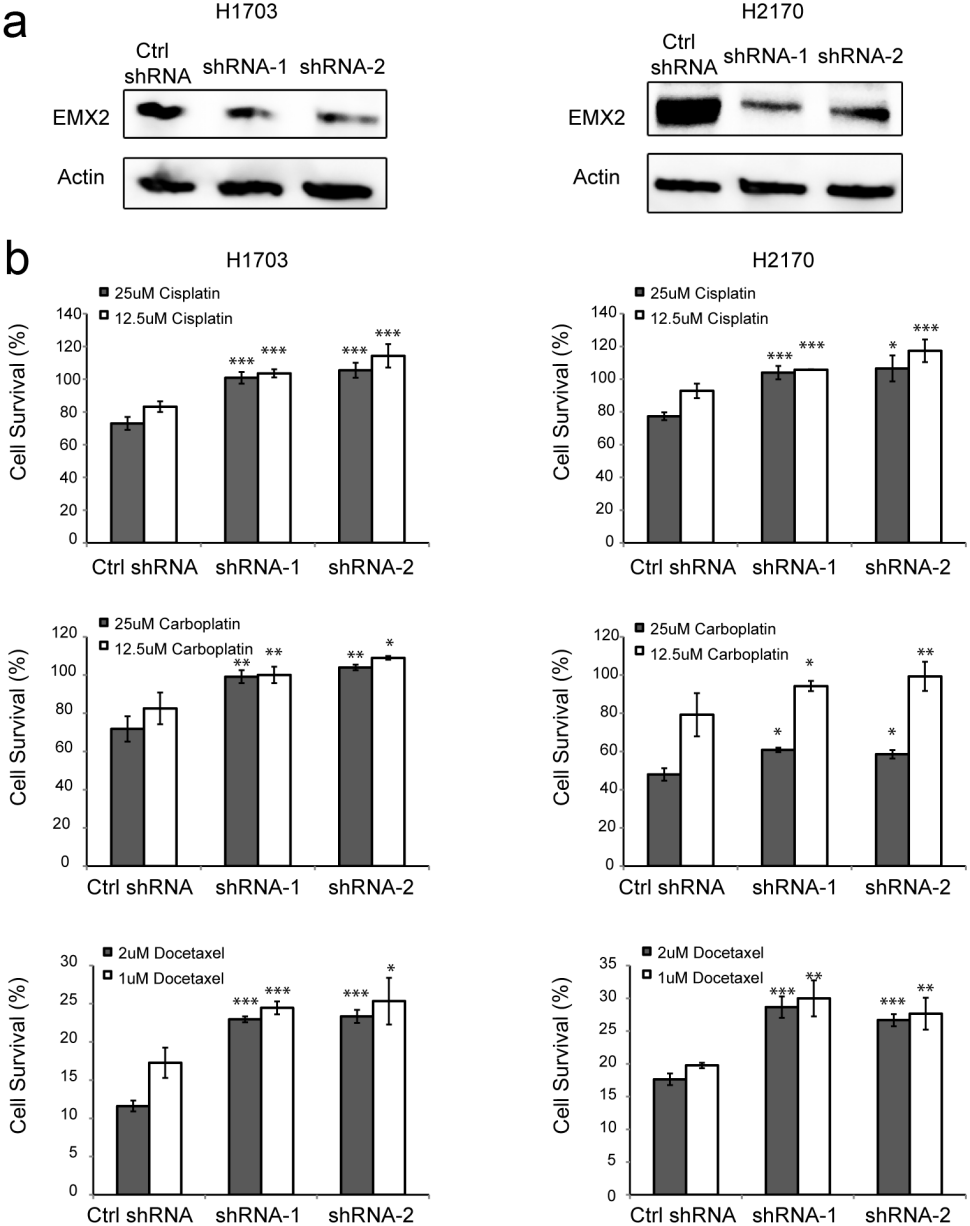
shRNA knock-down of EMX2 expression promotes chemo-resistance in lung SCC cells. (a) Western blots of EMX2 expression in lung SCC cell lines H1703 and H2170 stably transfected with different EMX2 shRNA constructs. Actin served as a loading control. (b) Cell survival (%) of stably transfected H1703 and H2170 cells after treatments with chemo drugs: cisplatin at 12.5 μM and 25 μM, carboplatin at 12.5 μM and 25 μM, and docetaxel at 1.0 μM and 2.0 μM, respectively. Cell survival was determined by MTS assays and measurements were normalized to that of H1703 or H1270 cells stably transfected with a non-silencing shRNA construct and treated with DMSO, which was set as 100%. Two-sided student’s *t*-test was performed between control shRNA and EMX2 shRNA expressing cell lines. A *p* value of 0.05 or less was indicated as *, 0.01 or less as **, and 0.001 or less as ***.

**Fig 4:**
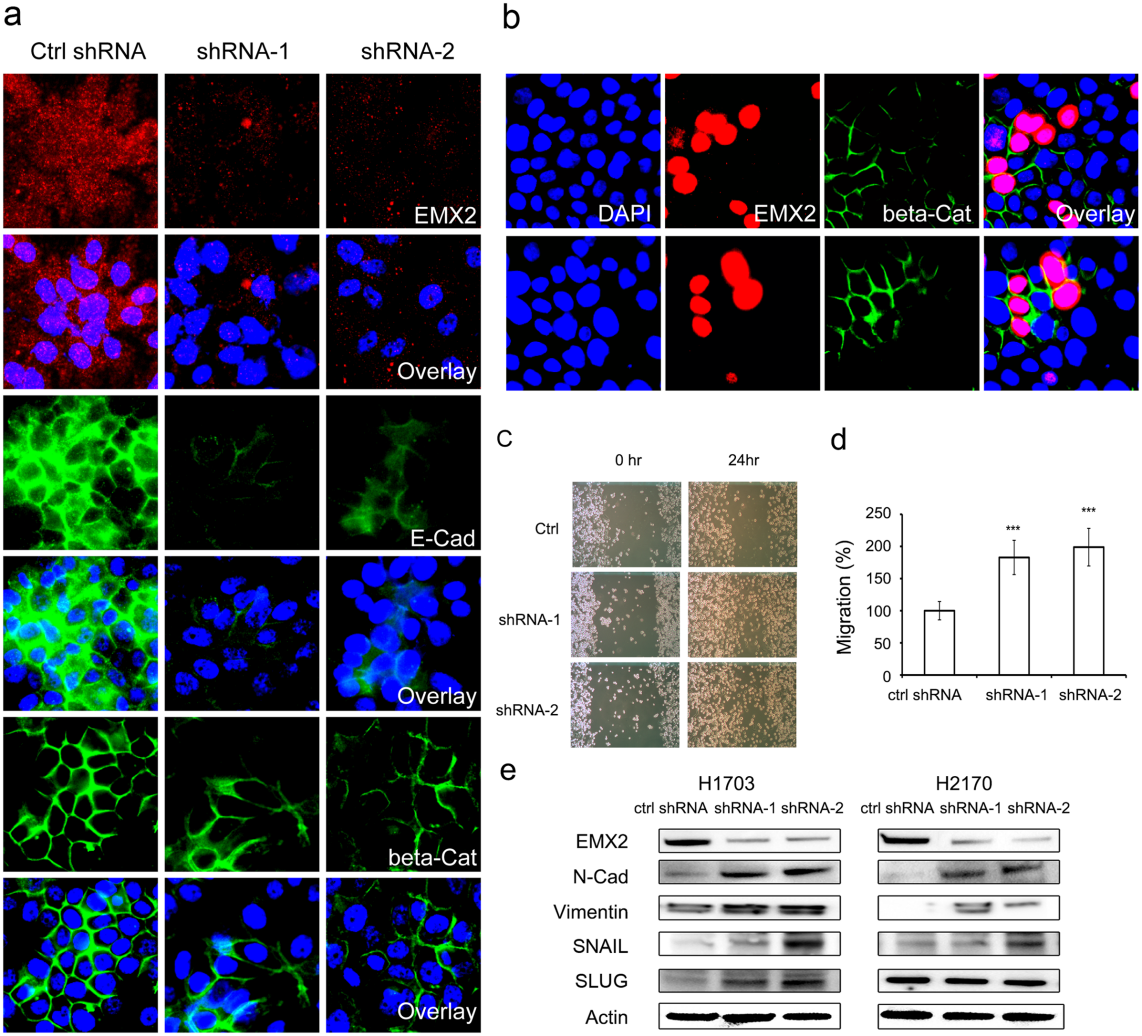
EMX2 may regulate EMT in lung SCC cell lines. (a) IF staining of EMX2 (red), E-Cadherin (green) and β-Catenin (green) in H2170 cells stably transfected with control shRNA or EMX2 shRNAs. (b) IF staining of EMX2 (red) and β-Catenin (green) in H1703 cells transiently transfected with EMX2 cDNA. Two representative fields were shown where the transfected cells had dramatic co-up-regulation of EMX2 and membrane β-Catenin, compared to the non-transfected cells in the fields. DAPI (blue) was used to stain nuclei of those cells for both panels (a) and (b). (c) Wound healing assays of lung SCC H2170 cells stably transfected with EMX2 shRNAs. Representative images shown at 0hr and 24hr were taken under a light microscope (100X). (d) Quantification of the wound healing assays. The migration distance of cells stably transfected with control shRNA was set as 100%. A *p* value of 0.001 or less was indicated as ***. (e) Western blot of EMT markers N-Cadherin and Vimentin and EMT regulators SNAIL and SLUG in lung SCC cell lines H1703 and H2170 stably transfected with different EMX2 shRNA constructs. Actin served as a loading control.

To investigate whether EMX2 expression could predict benefit from adjuvant chemotherapy, we analyzed IHC staining of EMX2 in these stage II/IIIA lung SCC specimens. We then established stable lung SCC cell lines after transfection with two different EMX2 shRNAs and subsequent G418 selection, and confirmed down-regulation of EMX2 expression in these stable transfectants (Fig 3a and Fig 4e). Next we treated these stable lines with three chemo drugs that are commonly used for the treatment of lung cancer, cisplatin, carboplatin, and docetaxel, respectively. We found that down-regulation of EMX2 expression by shRNA made H1703 and H2170 cells significantly more resistant to all three chemo treatments with elevated IC_50_ values compared to the control cells stably transfected with a non-silencing shRNA construct (Fig 3b, S1 Table). These results support our observation that EMX2 is associated with improved survival in lung SCC patients receiving adjuvant chemotherapy (Fig 2).

### EMX2 may regulate Epithelial-to-Mesenchymal Transition (EMT) in lung SCC

**Fig 5:**
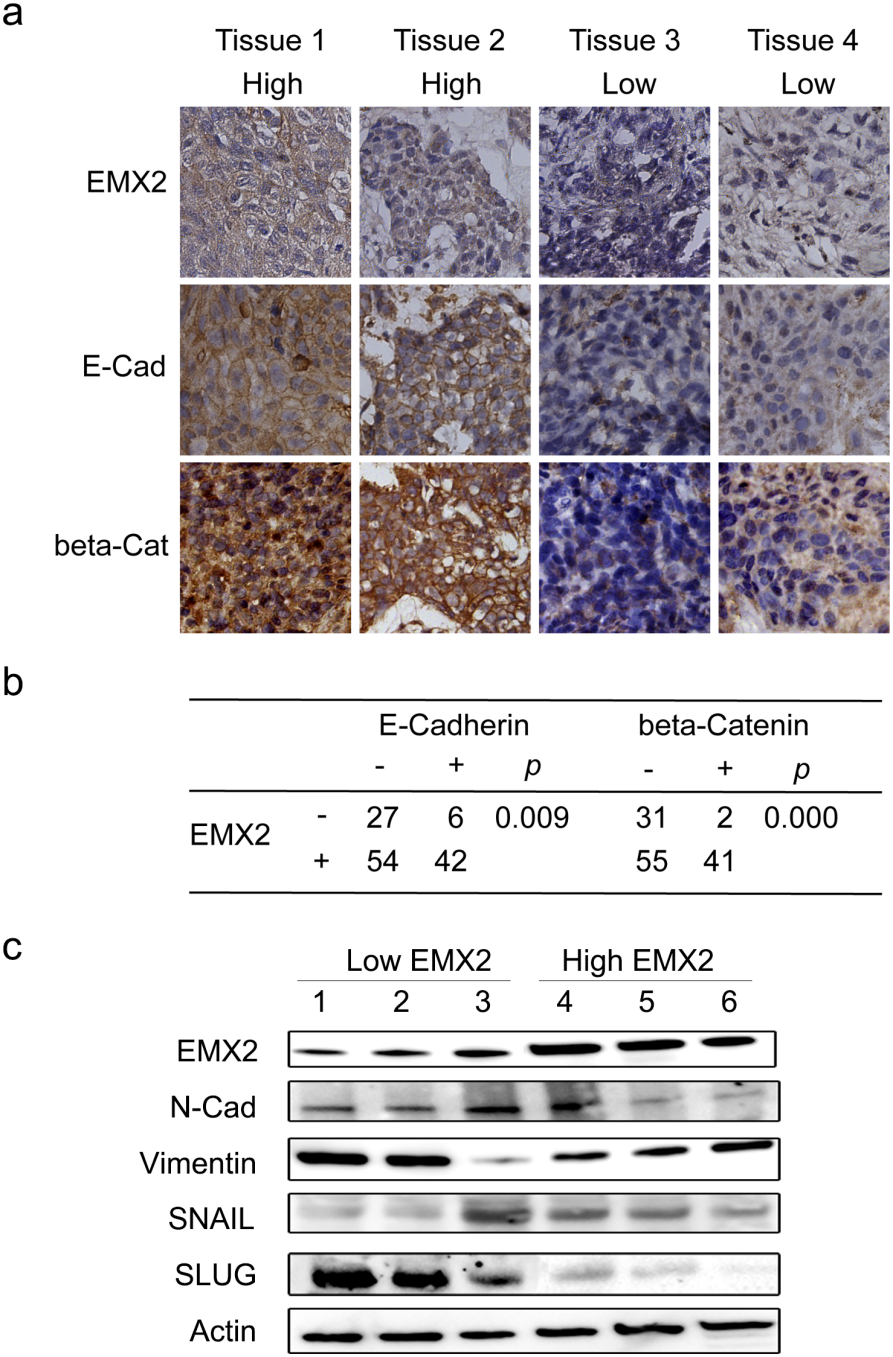
(a) IHC staining of EMX2 and EMT markers in lung SCC tissue samples. 129 specimens in Cohort-1 were available for staining for all three proteins: EMX2, E-Cadherin and β-Catenin. Two representative specimens with positive EMX2 expression (tissues 1 and 2, labeled as High) and two representative ones with negative EMX2 expression (tissues 3 and 4, labeled as Low) are shown. (b) Quantification of IHC staining in Cohort-1. Kappa test was also performed with IHC scores of 1–3 grouped as “+”, 0 as “-”. Kappa test’s *p* values were included in the table. (c) Western blot of EMT markers N-Cadherin and Vimentin, and EMT regulators SNAIL and SLUG in six tissue samples. Three representative samples with low EMX2 expression and three with high EMX2 expression. Actin served as a loading control.

EMT has been shown to be associated with poor prognosis and chemo-resistance in different tumor models (21–23). Our data demonstrated that EMX2 was down-regulated in lung SCC and this down-regulation was associated with chemo-resistance in lung SCC cells. Therefore, we decided to examine whether EMX2 was associated with EMT in lung SCC. Interestingly, we observed that hallmarks of EMT, E-Cadherin and the membrane-bound β-Catenin were significantly down-regulated when EMX2 expression was silenced by shRNAs in lung SCC cells assessed via immunofluorescence (IF) (Fig 4a). On the other hand, transfection of EMX2 cDNA into lung SCC cells resulted in up-regulation of the membrane-bound β-Catenin in the cells over-expressing EMX2 (Fig 4b). The association between EMX2 and EMT was also confirmed by cell migration assays with lung SCC cells stably transfected with two different EMX2 shRNA constructs. We observed that cell migration was significantly promoted at 24hr when endogenous EMX2 expression was silenced by the shRNAs in these cells (Fig 4c and 4d, *p* < 0.001). Cell proliferation was comparable between the control and shRNA cell lines (data not shown). In addition, immunofluorescence (IF) expression analyses of EMX2, E-Cadherin and membrane-bound β-Catenin in 129 lung SCC tissue samples in Cohort-1 revealed that EXM2 expression was positively correlated with that of E-Cadherin (*p* = 0.009) and membrane-bound β-Catenin (*p* = 0.000) in these lung SCC specimens examined (Figs 5a and 4b). The correlation between EMX2 and EMT was further confirmed with EMT markers N-Cadherin and Vimentin in both cultured cells (Fig 4e) and tissues (Fig 5c). EMT transcription factors SNAIL and SLUG were also examined, and the correlation between EMX2 and SNAIL or SLUG was not clear, which needs further investigation (Fig 4e and Fig 5c).

**Fig 6:**
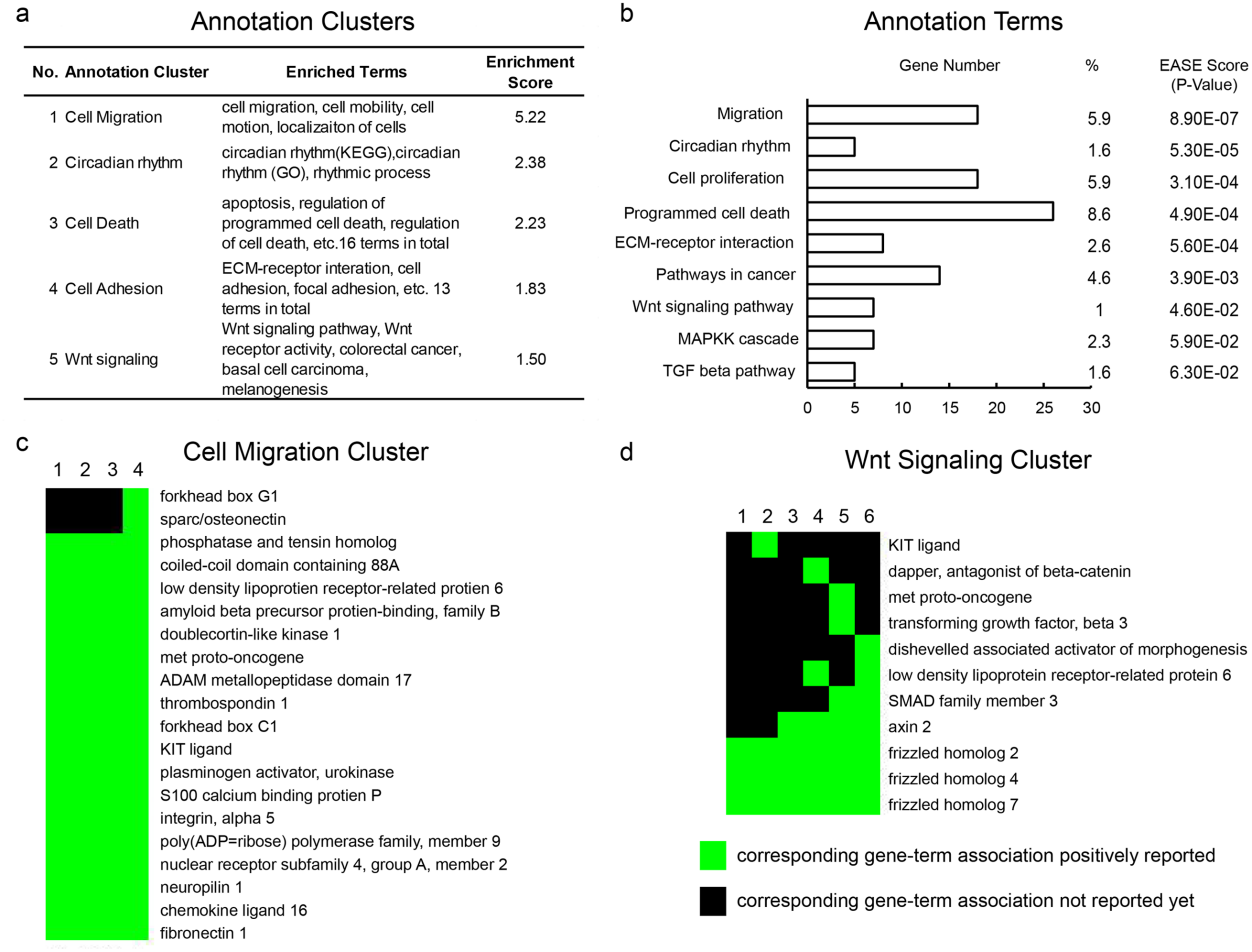
Functional annotation clustering of microarray data of H2170 cells stably transfected with EMX2 or empty vector control. Genes that exhibited more than 2-fold expression changes were analyzed with the Functional Annotation Clustering Tool of DAVID Bioinformatics Database by comparing with the Gene Ontology (GO) database and the KEGG_Pathway database. (a) Summary of functional annotation clusters. The higher the Enrichment scores, the more enriched. (b) Summary of enriched terms. Representative terms were selected among those that are similar. % is the percentage of involved gene / total gene numbers of a particular term. EASE score is a modified Fisher Exact *p*-value. Usually a *p*-value is equal or smaller than 0.05 to be considered strongly enriched in the annotation categories. (c) The migration annotation cluster. Every column represented a gene term, and the four terms were 1-GO:0016477-cell migration, 2- GO:0051674-localization of cell, 3-GO:0048870-cell motility, 4-GO:0006928-cell motion. (d) The Wnt pathway annotation cluster. Every column represented a gene term, and the six terms were 1-GO:0042813-Wnt receptor activity, 2-hsa04916-Melanogenesis, 3-hsa05217-Basal cell carcinoma, 4-GO:0016055-Wnt receptor signaling pathway, 5-hsa05210-Colorectal cancer, 6-hsa04310-Wnt signaling pathway. The green squares represent corresponding gene-term association positively reported, and the black squares represent corresponding gene-term association not reported yet.

In order to explore the underlying molecular mechanism of EMX2 in lung SCC, we performed a microarray analysis of H2170 cells that were stably transfected with EMX2 or empty vector control. There were 409 genes which exhibited more than 2-fold expression changes. We analyzed functional annotation with the Functional Annotation Clustering tool of DAVID Bioinformatics Database [24, 25] by comparing results with the Gene Ontology (GO) database and the KEGG_Pathway database (Fig 6). The most enriched annotation cluster with an Enrichment Score (ES) of 5.22 contained four enriched annotation terms, namely ‘cell migration, cell motion, cell motility, and localization of cells’. The annotation term ‘cell migration’ had an EASE score (a modified Fisher Exact *p*-value) of 8.9E-7, suggesting cell migration genes were strongly enriched in the list of genes upon EMX2 overexpression (Fig 6a and 6b). Genes in the cell migration annotation cluster are shown in Fig 6c. Other enriched annotation clusters included cell adhesion and cell death. Taken together, these results suggest, for the first time, that EMX2 may play an important role as a novel regulator of EMT and may, by this role, mediate chemo-resistance in lung SCC.

## Discussion

Aberrant homeobox gene expressionhas been reported in several types of cancers [26, 27], suggesting the significant role of homeobox genes in oncogenesis. However, fundamental questions still need to be fully addressed, such as the molecular mechanisms that drive the aberrant expression, downstream targets and signaling pathways that promote oncogenesis. To investigate the role that homeobox genes play in oncogenesis, our group and collaborators have recently identified EMX2 as being epigenetically down-regulated and serving as a putative novel tumor suppressor in lung adenocarcinomas and gastric cancer [16–18]. In the present study, we investigated whether EMX2 was also down-regulated and what role it might play in human lung SCC.

Our results demonstrated a significant decrease in EMX2 expression in primary lung SCC tissue samples when compared to their adjacent normal tissues, similar to that observed in lung adenocarcinomas [17]. Moreover, when we correlated the EMX2 expression levels with patient clinical outcomes, we found that positive EMX2 expression was significantly associated with improved overall survival in stage I lung SCC patients who received surgery, but without either neo-adjuvant or adjuvant chemotherapy. This suggests that EMX2 is a putative independent novel prognostic marker for stage I lung SCC patients. Together with our observations that high EMX2 expression levels were significantly associated with improved overall survival in early stage lung adenocarcinomas [18], it appears that EMX2 may play similar roles in both subtypes of human NSCLC.

We have previously shown that EMX2 suppresses lung cancer cell proliferation and sensitizes lung adenocarcinoma cells to cisplatin *in vitro* [17]. In this study, lung cancer SCC patients with positive EMX2 staining significantly benefited from adjuvant chemotherapy in two independent cohorts. In contrast, patients with negative EMX2 staining did not benefit from adjuvant chemotherapy. This result suggests that EMX2 may serve as a novel predictive marker for sensitivity to adjuvant chemotherapy in stage II/IIIA lung SCC patients. To support our finding, we demonstrated an increase in resistance of lung SCC cell lines to routinely used chemo drugs by down-regulating endogenous EMX2 expression. Our microarray analysis shows EMX2 overexpression strongly enriches annotation clusters of genes related to apoptosis. This suggests that the mechanism for chemotherapy sensitivity is possibly due to an increased tendency for apoptosis in EMX2 positive cells. One mechanism by which tumor cells evade apoptosis when treated with cisplatin is by up-regulating DNA repair mechanisms. In cells that overexpress EMX2, a small amount of cisplatin-induced DNA damage may be sufficient to trigger apoptotic pathways instead of being simply repaired by the tumor cells. There is little in the literature regarding EMX2, cellular stress response and apoptotic pathways. More research is needed to clarify this relationship.

Our finding that EMX2 is a predictive marker for sensitivity to adjuvant chemotherapy was validated by two well-controlled independent cohorts in a double-blinded manner. One limitation of this study, however, is the number of patient samples analyzed. A larger number of patient samples need to be examined to further validate our observation. Once confirmed, a prospective multi-center clinical trial would be the next logical step to provide more complete support for its clinical utility to better stratify early-mid stage lung SCC patients for benefits from adjuvant chemotherapy. Nevertheless, our results demonstrate, for the first time, the prognostic and predictive significance of EMX2 expression in lung SCC, suggesting exigencies for finding reliable prognostic and/or predictive markers for lung SCC. Because clinical evidence has shown that adjuvant therapy could be beneficial even for early-stage NSCLC, more meaningful stratification is necessary after surgical resection.

The biological mechanisms underlying the functional role of EMX2 as a prognostic and predictive marker in lung SCC biology are still unclear. Because EMT is known to enable cancer cells to invade surrounding tissues and generate distant metastases [20], and has been shown to be associated with poor prognosis and chemo-resistance in different tumor models [21–23], it is tempting to speculate that EMX2 is involved in the regulation of EMT in lung SCC. Indeed, we observed that EMX2 expression was significantly associated with expression of several EMT markers in both lung SCC cell lines and tissue samples. In addition, shRNA knockdown of endogenous EMX2 expression in lung SCC cells promoted cell migration and down-regulated EMT markers, while over-expressing EMX2 up-regulated these EMT markers, and affected the expression profiles of cell migration and cell adhesion annotation clusters. Whether EMX2 directly or indirectly regulates expression of the EMT markers in lung SCC still needs to be elucidated. The functional annotation analysis showed enrichment in the annotation term ‘pathways in cancer’ with 14 genes. The top three enriched pathways were Wnt (*p* = 0.046), MAPK (*p* = 0.059) and TGF-beta (*p* = 0.063) signaling pathways. The Wnt pathway was among the most enriched annotation clusters with a *p*-value <0.05 (Fig 6). In addition, silencing EMX2 has been suggested to aberrantly activate Wnt signaling in lung adenocarcinoma [17], and some EMT markers are downstream targets of Wnt pathway, such as E-cadherin [28]. Therefore, we postulate that Wnt signaling may play a role in mediating EMX2 function in the regulation of EMT in lung SCC. Not only is the role of the Wnt pathway known to be integral to embryo lung development, cell fate and differentiation but also deregulated Wnt signaling is well documented in lung cancer [13–16, 29]. Thus silencing EMX2, which causes aberrant Wnt signaling, might negatively impact squamous differentiation in SCC ultimately leading to tumor development, poor prognosis and resistance to therapy [30]. Further, it is possible that EMX2 may regulate other signaling pathways, such as the TGF-beta and MAPK pathways. An extensive analysis is necessary to elucidate the molecular mechanisms underlying EMX2 as a prognostic and predictive marker and a candidate EMT regulator in lung SCC.

## Supporting Information

S1 Table Summary of IC50 values of EMX2 silencing cell lines. Efficacy (IC_50_) of chemo drugs cisplatin or carboplatin in H1703 and H2170 cells stably transfected with EMX2 shRNAs or non-silencing shRNA construct (control) was determined by MTS assays. Cells were treated with corresponding chemo drugs at 7 different concentrations (2.5 μM, 5.0 μM, 10.0 μM, 20.0 μM, 30.0 μM, 40.0 μM, 50.0 μM) for 72 hours to obtain a dose-respond curve in order to determine IC_50_ values. Two-sided student’s t-test was performed between control shRNA and EMX2 shRNA lines. The data showed that EMX2 shRNA transfection significantly increased resistance of H1703 and H2170 cells to cisplatin or carboplatin treatment with elevated IC_50_ values compared to the control shRNA transfection in those cells (*p* values < 0.05).
(DOCX)
