# Supplementary figures and images for: EMX2 Is a Predictive Marker for Adjuvant Chemotherapy in Lung Squamous Cell Carcinomas
Source: PLoS One. 2015 Jul 1;10(7):e0132134. doi: 10.1371/journal.pone.0132134 (PMC4488446; doi:10.1371/journal.pone.0132134)

S1 Table. Summary of IC50 values of EMX2 silencing cell lines.


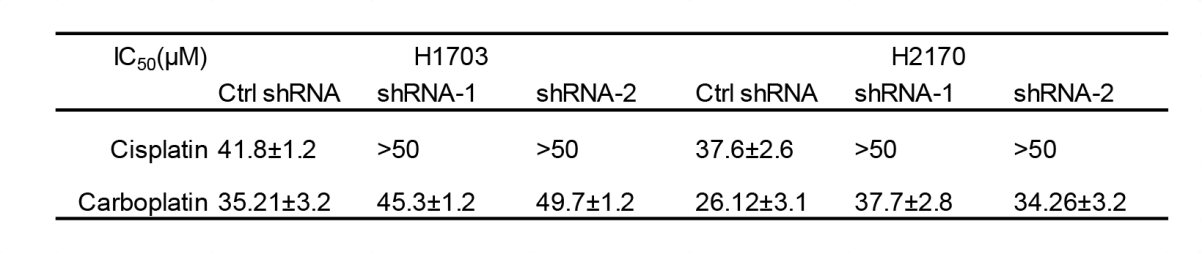

Supplement: S1 Table — Efficacy (IC50) of chemo drugs cisplatin or carboplatin in H1703 and H2170 cells stably transfected with EMX2 shRNAs or non-silencing shRNA construct (control) was determined by MTS assays. Cells were treated with corresponding chemo drugs at 7 different concentrations (2.5 μM, 5.0 μM, 10.0 μM, 20.0 μM, 30.0 μM, 40.0 μM, 50.0 μM) for 72 hours to obtain a dose-respond curve in order to determine IC50 values. Two-sided student’s t-test was performed between control shRNA and EMX2 shRNA lines. The data showed that EMX2 shRNA transfection significantly increased resistance of H1703 and H2170 cells to cisplatin or carboplatin treatment with elevated IC50 values compared to the control shRNA transfection in those cells (p values < 0.05). (DOCX) [file pone.0132134.s001.docx]
